# Effects of *Bacillus subtilis* WB60 and *Lactococcus lactis* on Growth, Immune Responses, Histology and Gene Expression in Nile Tilapia, *Oreochromis niloticus*

**DOI:** 10.3390/microorganisms8010067

**Published:** 2020-01-01

**Authors:** Seonghun Won, Ali Hamidoghli, Wonsuk Choi, Youngjin Park, Won Je Jang, In-Soo Kong, Sungchul C. Bai

**Affiliations:** 1 Department of Marine Bio-materials and Aquaculture, Feeds and Foods Nutrition Research Center (FFNRC), Pukyong National University, Busan 608-737, Korea; ks0sk@naver.com (S.W.); alihamid@pukyong.ac.kr (A.H.); thm622@naver.com (W.C.); 2 Faculty of Biosciences and Aquaculture, Nord University, Universitetsallen 11, 8049 Bodo, Norway; xxmain@naver.com; 3 Department of Biotechnology, Pukyong National University, Busan 608-737, Korea; jangwj9914@naver.com

**Keywords:** probiotic, *Bacillus*, *Lactococcus*, Nile tilapia, growth, immunity

## Abstract

An eight-week feeding trial was conducted to evaluate the effects of a basal control diet (CON), *Bacillus subtilis* at 10^7^ (BS_7_) and at 10^8^ CFU/g diet (BS_8_), *Lactococcus lactis* at 10^7^ CFU/g (LL_7_) and at 10^8^ CFU/g diet (LL_8_), and oxytetracycline (OTC) at 4 g/kg diet on Nile tilapia. Fish with initial body weight of 2.83 ± 0.05 g (mean ± SD) were fed two times a day. Weight gain, specific growth rate, feed efficiency, protein efficiency ratio and lysozyme activity of fish fed BS_8_, LL_8_ and LL_7_ diets were significantly higher than those of fish fed CON diet (*p* < 0.05). Superoxide dismutase and myeloperoxidase activity of fish fed BS_8_, LL_8,_ BS_7_, LL_7_ and OTC diets were significantly higher than those of fish fed CON diet. Intestinal villi length and muscular layer thickness of fish fed BS_8_, LL_8_ and LL_7_ diets were significantly higher than those of fish fed CON and OTC diets. Also, heat shock protein 70 (HSP70), interleukin (IL-1β), interferon-gamma (IFN-γ) and tumour necrosis factor (TNF-α) gene expression of fish fed BS_8_ and LL_8_ diets were significantly higher than those of fish fed CON diet. After 13 days of challenge test, cumulative survival rate of fish fed BS_8_ and LL_8_ diets were significantly higher than those of fish fed CON, BS_7_ and OTC diets. Based on these results, *B. subtilis* and *L. lactis* at 10^8^ (CFU/g) could replace antibiotics, and have beneficial effects on growth, immunity, histology, gene expression, and disease resistance in Nile tilapia.

## 1. Introduction

Aquaculture is one of the fastest growing food-producing sectors worldwide [1]. Along with the escalating demand for fish, the world aquaculture has become intensified with minimum land and water usage. However, the intensive aquaculture is confronted with problems that have led to concerns about fish welfare regarding growth problems, mortality and disease outbreaks [2,3]. As a consequence, most farmers have been using antibiotics prophylactically to overcome these problems [4]. Antibiotics are generally used to control disease and promote growth in the aquaculture industry. However, the use of antibiotics in aquaculture may alter the intestinal microbiota and give rise to resistant bacteria, which could be harmful to aquatic organisms [5]. On account of these reasons, several previous studies have been carried out on feed additives to replace antibiotics in aquaculture [6,7,8].

Recently, the potential of probiotics to reduce the use of antimicrobials and antibiotics in disease control has been highlighted. Probiotics have been used as an alternative environment-friendly strategy to develop sustainable aquaculture and have been defined in several ways; as live and viable microbial food supplements that provide beneficial effects to fish in terms of growth performances, immune responses, intestinal microbial balance and digestive enzyme activities [9,10]. Probiotics such as *Bacillus subtilis* [11], *Baillus licheniformis* [12], *Lactococcus lactis* [13] and *Micrococcus luteus* [14] have been identified beneficial for aquaculture. Specially, *B. subtilis* is a common probiotic and it has been reported that 10^7^ and 10^8^ CFU/g of this bacteria can enhance growth and immune while improving gastrointestinal tract of fish [11]. Also, 10^8^ CFU/g *lactococcus lactis* has shown to improve disease resistance and enhance immune responses in Nile tilapia [13]. The studies on the effects of these probiotics on different fish and shrimp species are still going on [14]. 

As it was mentioned before, a number of properties are required for the description of probiotics. Gatesoupe [15] reported that survival and colonization in the gastrointestinal tract (GI) are important to recognize the characterization of probiotics. Furthermore, Balcázar et al. [10] suggested the application of highly viable probiotics in the GI and reported that using the probiotics isolated from the host would help to ensure stability and survival. Studies on probiotic isolation from GI from different fish species have been continuously conducted and this method was recognized as a way for selecting efficient bacteria [11,12,16].

Therefore, in line with the dearth of knowledge on dietary probiotics in freshwater aquaculture, the present study was conducted to investigate effects of different dietary probiotics with different levels to replace dietary antibiotics in Nile tilapia, *Oreochromis niloticus.*

## 2. Materials and Methods

The study was conducted under the guidelines of the Animal Ethics Committee Regulations, No.18-0125 issued by the Pukyong National University, Busan, Korea.

### 2.1. Probiotic Conditions

The probiotic strains tested in this study as antibiotic replacers were *Bacillus subtilis* and *Lactococcus lactis*. Among these probiotics, *B. subtilis* was isolated from the intestine of juvenile Japanese eel and identified as *B. subtilis* WB60 according to Lee et al. [11]. This probiotic was incubated at 30 °C for 72 h in LB broth (Sigma-Aldrich, St. Louis, MO, USA) and measured at 600 nm optical density (OD_600_) using spectrophotometer. Also, *L. lactis* was grown in MRS broth at 36 °C for 48 h following Xia et al. [13]. Two probiotics were washed in sterile saline and the concentration of the final suspension was calculated at 1 × 10^7^ and 10^8^ CFU/g in the diets.

### 2.2. Experimental Diets

Feed formulation and proximate composition of the basal diet is shown in Table 1. A basal control diet without supplementation of probiotics (CON), and five other diets by supplementing *B. subtilis* (BS_7_) and *L. lactis* (LL_7_) at 1 × 10^7^ CFU/g diet, *B. subtilis* (BS_8_) and *L. lactis* (LL_8_) at 1 × 10^8^ CFU/g diet, and oxytetracycline (OTC) at 4 g/kg diet were prepared. According to manufacturer’s instructions (Samyang anipharm Co. LTD. Seoul, Korea) and previous studies on antibiotic replacement, it was suggested that 0.4–0.5% OTC should be used in the diet to maintain effective pathogen protection during the experimental period [17,18]. 

Tuna by-product and soybean meal were served as the major protein sources, soybean oil as the lipid source, while wheat flour and corn starch as the carbohydrate sources. The preparation and storage of experimental diets were followed as described by Bai and Kim [19]. Briefly, all the dry ingredients were measured and mixed in an electric mixing machine, followed by the addition of corn oil and water until a dough was formed. Experimental diets were pelleted using a laboratory pelleting machine with a 1 mm diameter module (Baokyong Cmmercial Co., Busan, Korea). The pellets were air-dried for 72 h and stored at −20 °C until use. 

### 2.3. Experimental Fish and Feeding Trial

The feeding trial was conducted at the Pukyoung National University and Feeds and Foods Nutrition Research Center (FFNRC), Busan, Korea. Juvenile Nile tilapia were obtained from the Docheon fishfarm (Gyeongsangnam-do, Korea) and were carried at FFNRC. Fish were fed a basal diet and acclimatized at the experimental condition for two weeks. Prior to the feeding trial, fish were examined for external abnormalities and feed was withheld for 24 h. At the begging of the experiment, fish with the initial body weight of 2.83 ± 0.05 g (mean ± SD) were distributed into 24 (40-L) rectangular tanks (20 fish/tank) receiving filtered freshwater at a constant flow (1.5 L/min) from the main tank. Each tank was randomly allocated to one of the three replicates of the eight dietary treatments. Fish were fed twice daily (9:00 and 18:00 h) at 3–4% of wet body weight/day for 8 weeks. Supplemental aeration was provided to maintain the dissolved oxygen. The temperature of aquarium was maintained at 27.0 ± 0.8 °C and pH remained at 7.50 ± 0.05 throughout the feeding trial. Uneaten feeds were cleaned by siphoning off after 2 h of feeding trial and the condition of tank were maintained by scrubbing once per week.

### 2.4. Sample Collection and Analysis

At the end of the feeding trial, feed was withheld from fish for 24 h. The number and weight of total fish in separated tanks were measured to calculate final body weight (FBW), weight gain (WG), feed efficiency (FE), specific growth rate (SGR), protein efficiency ratio (PER) and survival. Nine fish from each diet (three fish per tank) were anesthetized then liver and viscera were removed for the calculation of hepatosomatic index (HSI) and viscerosomatic index (VSI), respectively. Additionally, three fish from each treatment group were anesthetized by tricaine methanesulphonate (MS 222, 100 ppm) to collect the blood samples. Serum samples were separated by centrifugation at 5000× *g* for 10 min and stored at −70 °C for the analysis of non-specific immune responses including superoxide dismutase (SOD), myeloperoxidase (MPO) and lysozyme, as well as biochemical parameters such as aspartate aminotransferase activity (AST), aminotransferase activity (ALT), total protein (TP) and glucose. Intestine of fish samples were used for the preparations of histological sections and measurements of digestive enzyme activity. The proximate composition of experimental diets and whole-body samples were analysed according to standard methods of AOAC [20]. 

### 2.5. Non-Specific Immune Responses Analysis

Lysozyme activity was determined by a turbidimetric assay. The sodium citrate buffer was prepared by mixing trisodium citrate dihydeate and citric acid (0.02 M, pH 5.52). Then, 20 µL of serum sample was added to *Micrococcus lysodeikticus* (0.2 mg/mL) suspension in a 0.05 M sodium phosphate buffer. The mixed suspension was placed in a 96-well plate and measured at 450 nm of wavelength between 0 and 30 min with a spectrophotometer. Meanwhile, SOD activity was carried out by the percentage of superoxide radical reaction inhibition rate with WST-1 (water soluble tetrazolium dye) substrate and xanthine oxidase using the SOD Assay Kit (Sigma-Aldrich, 19160, St. Louis, MO, USA) following the manufacturer’s instructions. The reaction was recorded at 450 nm absorbance by spectrophotometer, after incubating for 20 min at 37 °C. The percent inhibition of enzyme was calculated by mg protein and expressed as SOD unit/mg. MPO activity was measured following Quade and Roth [21]. Briefly, serum (20 µL) was diluted with HBSS (Hanks Balanced Salt Solution) without Ca^2+^ or Mg^2+^ (Sigma-Aldrich) and placed in each well of a 96-well plate. 35 µL of 3, 3′, 5, 5′ tetramethylbenzidine hydrochloride (TMB, 20 mM; Sigma-Aldrich) and H_2_O_2_ (5 mM) were subsequently added. The colour change reaction after 2 min was completed by supplementing 35 µL of 4 M sulphuric acid. Finally, the optical density was read at 450 nm in a spectrophotometer.

### 2.6. Real-Time PCR

Five fish per experimental diets were used for sample analysis after being anesthetized. Total RNA was extracted from mid intestine (50 mg) of fish using RiboEx™ (GeneALL, Seoul, Korea) following standard procedure (Riboclear plus, GeneAll, Korea). RNA concentration (ng/μL) and purity (OD 260:280) was quantified with nanodrop measurement (Thremo Fisher Scientific, Waltham, MA, USA). The cDNA was synthesized from 1 μg of RNA using manufacturers’ instructions of cDNA synthesis Kit (Takara, Japan). RNA isolation and preparation of cDNA by 1 μg of RNA was performed following Hasan et al. [22]. Then, primer and target gene were prepared by BIONICS company (Seoul, Korea; Table 2). Relative RNA level of the target genes (IL-1ß, TNF-α, IFN-γ and HSP90) were evaluated and calculated using endogenous β-actin RNA level.

### 2.7. Histomorphology of the Intestine

The mid-intestinal samples were collected from experimental groups (*n* = 5) and were preserved in 10% neutral buffered formalin, dehydrated in a graded ethanol series and embedded in paraffin according to standard histological process. Sections series of 6 μm were made with a microtome and stained with hematoxylin and eosin (H&E). The evaluation of villi height (VL) and muscular thickness (MT) was observed using light microscope (AX70 Olympus, Tokyo, Japan) equipped with scientific digital camera for microscopy (DIXI Optics, Daejeon, Korea) and processed by image analysis software (Image J 1.32j, National Institute of Health, Bethesda, MD, USA). 

### 2.8. Challenge Test

*Aeromonas hydrophila* is a well-known pathogen that generally causes disease in Nile tilapia [23]. The pathogenic bacterium, *A. hydrophila* KCTC2358, was received from the Department of Biotechnology, Pukyong National University, Busan, Republic of Korea. At first, bacteria was grown in 10 mL brain heart infusion (BHI; Becton, Dickinson and Company, Baltimore, MD, USA) broth and incubated at 37 °C for 24 h in a shaking incubator. Growth of *A. hydrophila* was observed by optical density of 600 (OD_600_ nm) using a spectrophotometer (Mecasys, Optizen, Korea), harvested by centrifugation and washed two times with 0.1 M PBS for further use. At the end of the experiment, eight fish from each tank were randomly collected and reorganized based on their previous dietary treatment groups in 12-L tanks and allowed to maintain for 24 h. Fish were injected intraperitoneally with 0.1 mL per fish of *A. hydrophila* KCTC2358 at 2 × 10^7^ CFU/mL (2 × LD_50_). Fish mortality was documented daily up to 13 days. Water temperature was controlled at 27 ± 0.5 °C (mean ± SD) during the bacterial challenge test. 

### 2.9. Enzyme Activities

The enzyme activities of trypsin, lipase and amylase were determined in the linear range by using enzyme assays kit (Biovision, Milpitas, CA, USA) and spectrophotometer. The preparation of each specific enzyme assays kit was carried out with standard solutions, substrate and assay buffer. Trypsin activity was measured after reading the absorbance of samples at 405 nm wavelength at 0 and 20 min. Lipase activity was prepared by reaction mix including assay buffer, DTNB probe and lipase substrate and was read by spectrophotometer at a wavelength of 412 nm for 20 min. Amylase activity was reacted with assay buffer and substrate mix, and was determined by absorbance of samples at a wavelength of 402 nm at 0 and 20 min. Specific enzyme activities were defined as the amount of enzyme that catalysed the conversion of 1 μmol of substrate per minute per mU (i.e., mU/mL).

### 2.10. Statistical Analysis

The obtained data were statistically analysed by using the one-way ANOVA (SAS Version 9.1, SAS Institute Inc., Cary, NC, USA) in order to test the effects of dietary probiotic treatments. When a significant treatment effect was observed, an LSD post hoc test was used to compare means. Treatment effects were considered to be significant at *p* < 0.05.

## 3. Results

### 3.1. Growth Performance and Whole Body Proximate Composition

The growth performances and survival of juvenile Nile tilapia fed two probiotic diets are shown in Table 3. Weight gain, specific growth rate, feed efficiency and protein efficiency ratio of fish fed BS_8_, LL_8_, LL_7_ and OTC diets were significantly higher than those of fish fed CON diet (*p* < 0.05), however, there were no significant difference among fish fed BS_8_, LL_8_, BS_7_, LL_7_ and OTC diets (*p* > 0.05). Fish survival varied from 90% to 96.7%, and no significant differences were shown among all the diets. On the other hand, there were no significant differences in hepatosomatic indices, viscerosomatic indices, and condition factor in all diets (*p* > 0.05). No significant differences were observed on body protein, lipid, moisture and ash contents among treatment groups (*p* > 0.05; Table 4).

### 3.2. Non-Specific Immune Responses

The non-specific immune responses including lysozyme activity (LYZ), superoxide dismutase (SOD) and myeloperoxidase (MPO) were presented in Table 5. LYZ of fish fed BS_8_, LL_8_, LL_7_ and OTC diets were significantly higher than those of fish fed CON diet (*p* < 0.05). SOD and MPO activity of fish fed BS_8_, LL_8,_ BS_7,_ LL_7_ and OTC diets were significantly higher than those of fish fed CON diet (*p* < 0.05).

### 3.3. Haematological Analysis

As shown in Table 6, there were no significant differences in haematological analysis regarding alanine aminotransferase activity (ALT), glucose and total protein among fish fed the experimental diets (*p* < 0.05). However, aspartate aminotransferase activity (AST) of fish fed OTC diet were significantly higher than those of fish fed the other diets (*p* < 0.05). 

### 3.4. Intestinal Histology

**Figure 1:**
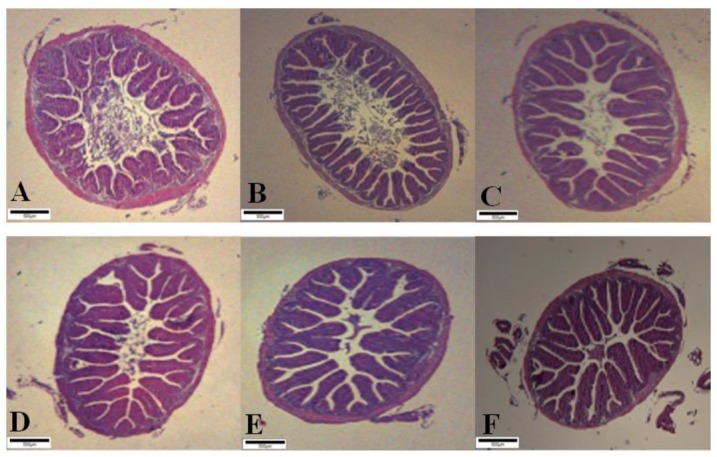
Histological sections of juvenile Nile tilapia intestine fed the experimental diets for 8 weeks. A-CON, basal diet; B-OTC, oxytetracycline; oxytetracycline at 4 g/kg; C-BS_7_, *Bacillus subtilis* at 1 × 10^7^ CFU/g; D- BS_8_, *Bacillus subtilis* at 1 × 10^8^ CFU/g; E- LL_7_, *Lactococcus lactis* at 1 × 10^7^ CFU/g; F-LL_7_, *Lactococcus lactis* at 1 × 10^8^ CFU/g. (Scale bar = 100 µm; Original magnification × 4).

The intestinal histology of Nile tilapia fed the experimental diets for 8 weeks is illustrated in Figure 1 and Table 7. Intestinal villi length of fish fed BS_8_ and LL_8_ diets were significantly higher than those of fish fed CON, OTC and BS_7_ diets (*p* < 0.05). However, there was no significant differences among fish fed BS_8_, LL_8_ and BS_7_ diets (*p* > 0.05). Muscular layer thickness of fish fed BS_8_, LL_8_ and LL_7_ diets were significantly higher than those of fish fed CON and OTC diets (*p* < 0.05). However, there were no significant differences among fish fed probiotics diets.

### 3.5. Immune-Related Gene Expressions

The immune-related gene expressions of the Nile tilapia intestine fed experimental diets are displayed in Table 8. Heat shock protein 70 (HSP70) levels of LL_8_ fed fish were significantly higher than those of fish fed CON, BS_7_ and OTC diets (*p* < 0.05). However, there were no significant difference among BS_8_, LL_8_ and LL_7_ groups (*p* > 0.05). The expression of interleukin (IL-1β) was higher in BS_8_ and LL_8_ fed groups compared to those of fish fed CON, BS_7_, LL_7_ and OTC diets (*p* < 0.05). On the other hand, tumour necrosis factor (TNF-α) and interferon-gamma (IFN-γ) expression in LL_8_ fed fish were significantly higher compared to CON, BS_7_ and OTC diets (*p* < 0.05), while they showed no significant differences among fish fed BS_8_ and LL_8_ diets (*p* > 0.05). 

### 3.6. Challenge Test

**Figure 2:**
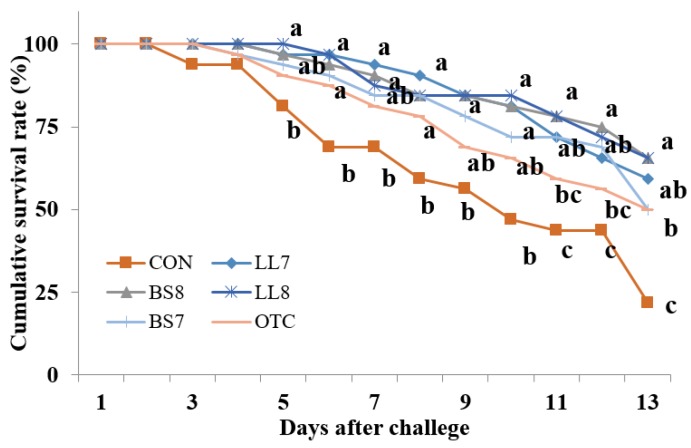
Cumulative survival rate of juvenile Nile tilapia fed the experimental diets with two probiotics for 8 weeks and experimentally challenged with *A. hydrophila* for 13 days. Each value represents mean of triplicate groups. Significant differences among means are indicated by different superscripts (*p* < 0.05). For information on diets refer to Table 3.

Cumulative survival rate of juvenile Nile tilapia against *Aeromonas hydrophila* for 13 days is provided in Figure 2. Fish mortalities initially occurred on the third day post injection. At the end of 13 days of challenge test, cumulative survival rate of fish fed BS_8_ and LL_8_ diets were significantly higher than those of fish fed CON, BS_7_ and OTC diets (*p* < 0.05). 

### 3.7. Enzyme Activities

The specific digestive activity of juvenile Nile tilapia is shown in Table 9. Trypsin activity of fish fed BS8, LL_8_ and LL_7_ diets were significantly higher than those of fish fed CON, BS_7_ and OTC diets (*p* < 0.05). Lipase and amylase activity did not differ among the all groups (*p* > 0.05).

## 4. Discussion

Antibiotics are common agents used as feed additives for growth and immune promotion through improved digestion and protection against pathogenic bacteria [24,25], but, as mentioned previously, excessive usage of antibiotics can induce the resistance of bacteria and cause severe environmental problems. Thus, research on the replacement of antibiotics is required for the sustainability of aquaculture. The role of probiotics in improving the growth and immune system has been considered and reviewed previously in aquaculture research studies [26,27]. Probiotics including *Bacillus* sp., *Lactococcus* sp., and *Micrococcus* sp. are commonly used as dietary additives where they can provide beneficial effects on the health condition, modulate intestinal homeostasis and promote gut health. To the best of our knowledge, the present study is first to compare potential probiotics such as *B. subtilis* and *L. lactis*, that could have beneficial effects on growth performance, health condition and disease resistance in Nile tilapia. 

Based on our findings, probiotics improved growth performance and feed efficiency of Nile tilapia, which is in agreement with previous observations suggesting growth enhancement by *B. subtilis* [11], *B. licheniformis* [12], *L. lactis* [13] and *M. luteus* [14]. The improved growth performance and feed-utilization of Nile tilapia fed probiotic diets could be due to enhanced intestinal histology and enzyme activities. Probiotics may have facilitated effective nutrient absorption by improved intestinal villi length, muscle layer thickness and trypsin activity which have been proven by our results shown in Figure 1 and Table 6. Xia et al. [13] reported that *L. lactis* can improve intestinal health and increase growth performance of Nile tilapia compared to CON diet. Furthermore, our results are similar with the finding of Liu et al. [28], who reported that *B. subtilis* supplementation could enhance the digestive enzyme activities of Nile tilapia and thus improve fish growth performance. Also, probiotic supplementation has led to increased digestive enzyme activities in shrimp *Penaeus vannamei* and improved growth performance [29]. In rainbow trout, *Onchorhynchus mykiss*, probiotic supplementation increased digestive enzyme activity and improved intestinal morphology which resulted in enhanced growth performance [30]. 

The non-specific immune parameters have been commonly used as indicators of health conditions in fish. Numerous studies [10,31,32,33] have investigated probiotics as important compounds in immune system modulation and disease prevention. Superoxide dismutase (SOD) and myeloperoxidase (MPO) are known to regulate the reactive oxygen species in numerous target cells and catalyse the conversion of hydrogen peroxide and superoxide radicals to normal oxygen [34,35]. In the current study, dietary probiotic groups improved SOD activity compared to CON group, but did not show significant differences with OTC. Moreover, MPO activity clearly showed a similar trend with SOD activity. Among the innate immune system parameters, lysozyme plays a key role regarding protection against host infections and constitutes a chemical and biological barrier of first defence against pathogens in fish [36]. In the present study, lysozyme activity of fish fed BS_8_, LL_8_, LL_7_ and OTC were significantly higher than those of fish fed CON diet. However, there were no differences among probiotics and OTC diets. Previous studies demonstrated probiotics can enhance immune responses such as SOD, MPO and lysozyme in Nile tilapia [37,38], lysozyme in rainbow trout [39], and SOD and lysozyme in grouper *Epinephelus malabaricus* [40]. One of the reasons for the improved immunity is the enhancement of phagocytic activity and reactive oxygen metabolites by macrophages. Observations from this study are in complete agreement with the findings of Newaj-Fyzul et al. [41] who found the number of leucocytes, respiratory burst and phagocytic activity increased significantly when *B. subtilis* was supplemented in rainbow trout diets. Ultimately, respiratory burst activity may have enhanced immunity by extracellular protection against pathogens [42,43]. Because of these reasons, numerous studies on probiotics have demonstrated that they are not only a potential antibiotic replacer in aquaculture but also elevate the growth, immune response and disease resistance of fish [44,45]. 

Interestingly, in the present study, antibiotic fed fish showed significantly higher aspartate aminotransferase (AST) than other treatment groups. Haematological parameters are known to determine health conditions and are important indicators of fish health. Especially, AST and ALT have been considered as biochemical parameter for fish under the expression of stress [46]. Previous studies suggested that antibiotics can influence the liver by hepatotoxic and lead to damage through physiological stress [47,48]. The AST value was not affected by probiotic supplementations in our study. 

Cytokines (e.g., IL-1β, TNF-α and IFN-γ) are signalling proteins that regulate a wide range of biological functions such as stress, immune responses, hematopoiesis and inflammation mainly through extracellular signalling [49]. Specially, they are secreted by cells of both innate and adaptive immune systems [50]. Interferon-γ, known as immune interferon, is primarily provided by activated T lymphocytes and possibly by natural killer cells. It has a pivotal mediator role for macrophage activation and has immunomodulatory properties [51]. The results of the present study showed that probiotic groups including BS_8_, LL_8_ and OTC had significantly higher IFN-γ expression compared to the CON, BS_7_ and LL_7_ groups. These results are similar to those of Xia et al. [13], which reported probiotic supplementation may influence the IFN-related gene, and improve disease resistance against *A. hydrophila.* IL-1β is an indicator of immune responses against pathogens and virus and participates in disease resistance, microbial invasion and tissue injury in fish, while TNF-α is mainly secreted by activated macrophages, and is involved in the immune response [51]. Observations of our study showed higher expression for IL-1β and TNF-α in fish fed LL_8_ diet compared to CON, BS_7_ and OTC, but they did not show any differences among BS_8_ and LL_8_ diets (Figure 2). This means that optimum probiotic levels in the diet of Nile tilapia can up regulate the expression of IL-1β and TNF-α genes. It was reported that the lymphoid cells in intestines were elevated by stimulation of probiotics [52]. Basically, administration of probiotics can activate the transcription levels of pro-inflammatory cytokines in fish [53]. In this study, pro-inflammatory cytokines were improved in probiotic groups compared to the CON. Liu et al. [54] demonstrated that the influence of dietary probiotics on the intestinal wall can stimulate the expression of cytokines. Furthermore, Gatesoupe [15] concluded that probiotic supplementation in the diet can improve antagonistic activity against pathogens and increase the resistance of the host. Heat shock protein 70 (HSP70) has been lately recognized as a potential immune response regulator [55]. In this study, probiotic-supplemented diets increased the expression of the HSP70 genes in the intestine of Nile tilapia, while LL_8_ fed fish were significantly higher than those of fish fed CON, BS_7_ and OTC diets. While, there were no significant differences among BS_8_, LL_8_ and LL_7_ groups. According to previous research, the probiotic bacterial communities can improve immune system in terms of HSP70 in Japanese eel [11] and Nile tilapia [13]. Overall gene expressions showed that optimum probiotics in Nile tilapia diets can improve immune responses and stimulated cytokine and protein enzymes. 

The intestinal histology including villi length and muscular layer thickness have been determined to estimate the gut condition of fish [56]. The villi length of Nile tilapia fed probiotic diets was significantly higher in the mid-intestine than those of fish fed CON and OTC diets. Specially, BS_8_ and LL_8_ diets showed highest length among the experimental groups, which was in agreement with the previous reports [11,13]. Likewise, muscular layer thickness was significantly higher in the mid-intestine of the probiotic supplemented groups compared to CON diet. Whereas, BS_8_ and LL_8_ diets significantly better than OTC diet. These results are in agreement with Lazado and Caipang [57], who reported that enhanced muscular layer thickness has been associated with probiotic administration. In addition, probiotics can modulate the physiological activities of gut mucosal cells. As previously stated, improvement of intestinal morphology increases the absorption of nutrients in surface area, and ultimately improves the growth performance. 

The cumulative survival rate against *A. hydrophila* for 13 days were significantly higher in Nile tilapia when fed dietary probiotics compared to CON. Previous studies have reported that probiotic diets such as *B. subtilis* and *L. lactics* elevate disease resistance of Nile tilapia against bacteria [13,58]. According to what was mentioned, probiotics may have improved non-specific immunity of Nile tilapia and thus enhanced disease resistance against *A. hydrophila* infection. Further, the results of the current study corroborated with the previous studies on the increased disease resistance by probiotic supplementations [59,60]. Besides, BS_8_ and LL_8_ diets showed potential effects as antibiotic replacers in Nile tilapia. The differing impact of probiotics with each concentration on disease resistance in tilapia can be related to differences in the antibiotic activities of specific probiotic strains.

Digestive enzyme activities are useful in estimating the potential of an organism to metabolize a given substrate, and also for establishment of feeding rhythms [61]. In the current study, trypsin activity of Nile tilapia was significantly influenced by BS_8_, LL_8_ and LL_7_ groups (Figure 2). According to Bowyer et al. [62], it was demonstrated that the response of the digestive enzyme activity is closely correlated with that of growth performance. Furthermore, probiotics secrete protease enzymes that can digest the peptide bonds in proteins and break down proteins into their constituent monomers and free amino acids; this process can benefit fish nutritionally [63]. In general, enzyme activity increases in fish when fed diets with probiotics, which was proven in common carp *Cyprinus carpio* [64], sea bass *Dicentrarchus labrax* [65] and Javanese carp *Puntius gonionotus* [66]. Meanwhile, lipase and amylase activities in our study were not affected by treatment diets. Some previous studies demonstrated that enzyme activities were not influenced by dietary probiotic supplementations in paddlefish *P. spathula* after 80 days [67] and in catla *C. catla* after 60 days [68]. The previous studies suggested that continuous feeding with excessive probiotics could inhibit the endogenous enzyme activities [67]. Apart from trypsin activity, further studies are needed to determine how probiotics improve enzyme activities such as lipase and amylase. 

## 5. Conclusions

The main goal of the present study was to evaluate the most effective dietary probiotic at a proper dosage in Nile tilapia *Oreochromis niloticus*. As mentioned above, dietary administration of *B. subtilis* (BS_8_) and *L. lactis* (LL_8_) at 1 × 10^8^ (CFU/g) could enhance growth performance, immune responses, intestinal morphology, disease resistance and gene expression in Nile tilapia. It has also been demonstrated that the use these probiotics, at the mentioned level, could offset the need for antibiotics in the culture of this fish. However, it should be considered that this experiment was conducted at laboratorial scale tanks using filtered water; for a more precise evaluation of these probiotics, farm scale experiments (using green water culture) are suggested. 

## Figures and Tables

**Table 1:** Formulation and composition (% dry matter) of the basal diet for Nile tilapia.

Ingredients	%
Tuna by-product ^1^	17.0
Squid liver powder ^2^	3.0
Blood meal ^2^	5.0
Soybean meal ^2^	31.5
Wheat gluten meal ^2^	6.1
Wheat flour ^2^	19.5
Corn starch ^2^	10.5
Soybean oil ^3^	2.4
Vitamin premix ^4^	1.0
Mineral premix ^5^	1.0
Calcium phosphate ^6^	2.0
Cellulose ^7^	1.0
***Proximate composition***	
Moisture	9.89
Crude protein	36.5
Crude lipid	6.19
Crude ash	6.24

^1^ Samhae Industrial Co., LTD. Gyeongju, Korea (Tuna by-product, crude protein: 47.5% and crude lipid: 23%). ^2^ The Feed Co., LTD. Seoul, Korea (Soybean meal, crude protein: 49.2% and crude lipid: 3.44%). ^3^ Jeil Feed Co. Hamman, Korea. ^4^ Contains (as mg/kg in diets): Thiamine mononitrate, 15; Rivoflavin, 30; Niacin, 150; dl-Calcium pantothenate, 150; Pyridoxine·HCl, 15; Biotin, 1.5; Folic acid, 5.4; Cobalamin, 0.06; Ascorbic acid, 300; Inositol, 150; Choline bitate, 3000; Retinyl acetate, 6; dl-α-Tocopherol acetate, 201; Menadion, 6. ^5^ Contains (as mg/kg in diets): Ca(IO)_3_, 0.0006; MgSO_4_·7H_2_O, 1379.8; NaCl, 437.4; NaSeO_3_, 0.00025; ZnSO_4_·7H_2_O, 226.4; MnSO_4_, 0.016; Fe-Citrate, 299; FeSO_4_, 0.0378; CuSO_4_, 0.00033; MgO, 0.00135.^6^ CaHPO_4_, Sigma-Aldrich Korea, Yongin, Korea; ^7^ United States Biochemical; Cellulose was used for energy source.

**Table 2:** Primers used to quantify relative gene expression.

Primers	Sense	Sequences
HSP90 ^1^	F	5′-CATCGCCTACGGTCTGGACAA-3′
	R	5′-TGCCGTCTTCAATGGTCAGGAT-3′
IL-1β ^2^	F	5′-CAAGGATGACGACAAGCCAACC-3′
	R	5′-AGCGGACAGACATGAGAGTGC-3′
IFN-γ ^3^	F	5′-AAGAATCGCAGCTCTGCACCAT-3′
	R	5′-GTGTCGTATTGCTGTGGCTTCC-3′
TNF-α ^4^	F	5′-GGAAGCAGCTCCACTCTGATGA-3′
	R	5′-CACAGCGTGTCTCCTTCGTTCA-3′
β-actin ^5^	F	5′-CCACACAGTGCCCATCTACGA-3′
	R	5′-CCACGCTCTGTCAGGATCTTCA-3′

^1^ HSP90, heat shock protein 90 (FJ207463.1). ^2^ IL-1β, interleukin (XM_003460625.2). ^3^ IFN-γ, interferon-gamma (XM_005448319.1). ^4^ TNF-α, tumour necrosis factor (JF957373.1). ^5^ β-actin (HQ386788.1).

**Table 3:** Growth performance and feed utilization of juvenile Nile tilapia fed the experimental diets for 8 weeks.

	Diets ^1^						Pooled SEM
	CON	OTC	BS_7_	BS_8_	LL_7_	LL_8_	
IBW (g) ^2^	2.85	2.83	2.83	2.80	2.82	2.85	0.01
FBW (g) ^3^	9.77 ^b^	10.6 ^a^	10.3 ^ab^	10.6 ^a^	10.6 ^a^	10.8 ^a^	0.15
WG (%) ^4^	242 ^b^	276 ^a^	264 ^ab^	277 ^a^	278 ^a^	279 ^a^	5.78
FE (%) ^5^	81.9 ^b^	93.5 ^a^	90.4 ^ab^	93.3 ^a^	92.7 ^a^	94.3 ^a^	1.90
SGR(%/day) ^6^	2.37 ^b^	2.54 ^a^	2.48 ^ab^	2.55 ^a^	2.56 ^a^	2.56 ^a^	0.03
PER ^7^	2.31 ^b^	2.58 ^a^	2.51 ^ab^	2.57 ^a^	2.53 ^a^	2.59 ^a^	0.04
HSI (%) ^8^	1.46	1.46	1.36	1.35	1.45	1.45	0.02
VSI (%) ^9^	7.39	7.70	7.75	7.43	7.12	7.84	0.11
CF ^10^	1.70	1.64	1.71	1.69	1.74	1.70	0.01
Survival (%) ^11^	90.0	96.7	96.7	95.0	95.0	95.0	1.00

^1^ Values are means ± SD of triplicate groups of fish. Values in each row with different superscripts are significantly different (*p* < 0.05). Diets: CON = the basal diet, refer to Table 1; BS_7_ = *B. subtilis* at 1 × 10^7^ CFU/g; LL_7_ = *L. lactis* at 1 × 10^7^ CFU/g; BS_8_ = *B. subtilis* at 1 × 10^8^ CFU/g; LL_8_ = *L. lactis* at 1 × 10^8^ CFU/g; OTC = oxytetracycline; oxytetracycline at 4 g/kg. ^2^ Initial body weight. ^3^ Final body weight. ^4^ Weight gain (WG, %) = ((final wt.−initial wt.) × 100)/initial wt. ^5^ Feed efficiency ratio (FE, %) = (wet weight gain / dry feed intake) × 100. ^6^ Specific growth rate (SGR, %) = ((log_e_ final wt.−log_e_ initial wt.) × 100)/days. ^7^ Protein efficiency ratio (PER) = (wet weight gain/protein intake). ^8^ Hepatosomatic index (HSI) = (liver wt. × 100)/body wt. ^9^ Visceralsomatic index (VSI, %) = (viscera wt. × 100)/body wt. ^10^ Condition factor = (wet weight/total length^3^) × 100. ^11^ Survival rate (%) = ((total fish−dead fish) × 100)/total fish. ^a,b^ Data with different superscripts are significantly different.

**Table 4:** Whole-body proximate composition (%, wet weight basis) of juvenile Nile tilapia fed the experimental diets for 8 weeks.

	Diets ^1^						Pooled SEM
	CON	OTC	BS_7_	BS_8_	LL_7_	LL_8_	
Moisture (%)	73.1	72.8	73.4	72.9	73.0	73.5	0.11
Protein (%)	17.0	17.1	17.6	16.9	17.0	17.4	0.15
Lipid (%)	5.44	5.59	5.78	5.69	5.79	5.95	0.07
Ash (%)	4.93	4.80	5.05	5.34	5.13	5.25	0.08

^1^ Values are means ± SD of triplicate groups of fish. Values in each row with different superscripts are significantly different (*p* < 0.05). For information on diets refer to Table 3.

**Table 5:** Non-specific immune responses of juvenile Nile tilapia fed the experimental diets for 8 weeks.

	Diets ^1^						Pooled SEM
	CON	OTC	BS_7_	BS_8_	LL_7_	LL_8_	
Lysozyme (U/mL)	1.23 ^b^	1.47 ^a^	1.68 ^ab^	1.69 ^a^	1.67 ^a^	1.80 ^a^	0.08
SOD ^2^	64.8 ^b^	81.2 ^a^	81.2 ^a^	83.5 ^a^	86.2 ^a^	81.1 ^a^	3.08
MPO ^3^	2.71 ^b^	3.68 ^a^	3.48 ^a^	3.94 ^a^	4.13 ^a^	3.90 ^a^	0.21

^1^ Values are means ± SD of triplicate groups of fish. Values in each row with different superscripts are significantly different (*p* < 0.05). For information on diets refer to Table 3. ^2^ SOD: Superoxide dismutase activity (% inhibition). ^3^ MPO: Myeloperoxidase activity (OD at 450 nm). ^a,b^ Data with different superscripts are significantly different.

**Table 6:** Haematological analysis of juvenile Nile tilapia fed the experimental diets for 8 weeks.

	Diets ^1^						Pooled SEM
	CON	OTC	BS_7_	BS_8_	LL_7_	LL_8_	
AST ^2^	66.7 ^b^	108 ^a^	78.3 ^b^	63.3 ^b^	56.7 ^b^	67.0 ^b^	7.50
ALT ^3^	5.33	5.33	5.33	5.33	6.00	5.33	0.11
Glucose (mg dL^−1^)	43.7	44.0	50.3	50.7	41.7	47.7	1.54
T-protein ^4^	2.71	3.23	2.87	2.53	3.00	2.73	0.10

^1^ Values are means ± SD of triplicate groups of fish. Values in each row with different superscripts are significantly different (*p* < 0.05). For information on diets refer to Table 3. ^2^ AST: Aspartate aminotransferase activity (U L^−1^). ^3^ ALT: Alanine aminotransferase activity (U L^−1^). ^4^ T-protein: Total protein (g dL^−1^). ^a,b^ Data with different superscripts are significantly different.

**Table 7:** Intestinal morphology of juvenile Nile tilapia fed the experimental diets for 8 weeks.

	Diets ^1^						Pooled SEM
	CON	OTC	BS_7_	BS_8_	LL_7_	LL_8_	
Villi length (μm)	202 ^d^	235 ^c^	253 ^b^	290 ^a^	273 ^ab^	285 ^a^	13.7
Muscular layer thickness (μm)	36.4 ^c^	46.2 ^b^	50.2 ^ab^	59.0 ^a^	53.3 ^a^	54.9 ^a^	3.24

^1^ Values are means ± SD of triplicate groups of fish. Values in each row with different superscripts are significantly different (*p* < 0.05). For information on diets refer to Table 3. ^a^^–^^d^ Data with different superscripts are significantly different.

**Table 8:** Intestinal gene expression levels of juvenile Nile tilapia fed the experimental diets for 8 weeks.

	Diets ^1^						Pooled SEM
	CON	OTC	BS_7_	BS_8_	LL_7_	LL_8_	
HSP70 ^2^	1.00 ^d^	6.42 ^bc^	3.79 ^cd^	7.51 ^ab^	6.83 ^ab^	9.68 ^a^	1.24
IL-1β ^3^	1.00 ^d^	5.37 ^c^	5.72 ^bc^	8.54 ^ab^	3.59 ^cd^	11.3 ^a^	1.48
IFN-γ ^4^	1.00 ^c^	8.22 ^a^	2.62 ^bc^	9.82 ^a^	3.78 ^b^	10.6 ^a^	1.65
TNF-α ^5^	1.00 ^c^	2.13 ^b^	2.17 ^b^	2.38 ^ab^	2.63 ^ab^	3.25 ^a^	0.30

^1^ Values are means ± SD of triplicate groups of fish. Values in each row with different superscripts are significantly different (*p* < 0.05). Diets refer to Table 3. ^2^ HSP70: Heat shock protein 70. ^3^ IL-1β: Interleukin. ^4^ IFN-γ: Interferon-gamma. ^5^ TNF-α: Tumour necrosis factor. ^a^^–^^d^ Data with different superscripts are significantly different.

**Table 9:** Enzyme activities of juvenile Nile tilapia fed the experimental diets for 8 weeks.

	Diets ^1^						Pooled SEM
	CON	OTC	BS_7_	BS_8_	LL_7_	LL_8_	
Trypsin (mU/mL)	0.73 ^b^	0.90 ^b^	0.83 ^b^	1.30 ^a^	1.54 ^a^	1.49 ^a^	0.14
Lipase (mU/mL)	3.07	3.11	3.32	3.43	3.01	3.58	0.09
Amylase (mU/mL)	3.73	3.75	3.75	3.66	3.69	3.74	0.02

^1^ Values are means ± SD of triplicate groups of fish. Values in each row with different superscripts are significantly different (*p* < 0.05). For information on diets refer to Table 3. ^a^^–^^d^ Data with different superscripts are significantly different.
